# IDH, Recursos Tecnológicos e Humanos para Diagnóstico e Tratamento das Malformações do Aparelho Circulatório no Brasil

**DOI:** 10.36660/abc.20200179

**Published:** 2021-07-15

**Authors:** Thais Rocha Salim, Thayanne Mendes Andrade, Carlos Henrique Klein, Gláucia Maria Moraes de Oliveira

**Affiliations:** 1 Universidade Federal do Rio de JaneiroRio de JaneiroRJBrasilUniversidade Federal do Rio de Janeiro, Rio de Janeiro, RJ - Brasil; 2 Universidade de VassourasVassourasRJBrasilUniversidade de Vassouras, Vassouras, RJ - Brasil; 3 Escola Nacional de Saúde PúblicaRio de JaneiroRJBrasilEscola Nacional de Saúde Pública, Rio de Janeiro, RJ - Brasil

**Keywords:** Anormalidades Congênitas, Anormalidades Cardiovasculares, Cardiopatias Congênitas/diagnóstico, Indicadores de Desenvolvimento, Mortalidade Infantil, Epidemiologia

## Abstract

**Fudamento:**

Em 2015, foram diagnosticados 2.368 portadores de malformação congênita (MC) por 100.000 nascidos vivos (NV) no mundo, uma taxa de 7,6%, dos quais 10,6% morreram no primeiro ano de vida, sendo 43% por malformações do aparelho circulatório (MAC), à semelhança do que ocorre no Brasil.

**Objetivo:**

Verificar a associação de diagnóstico de MAC ao nascimento e morte por MAC no primeiro ano de vida com índice de desenvolvimento humano (IDH) e recursos tecnológicos e humanos para o diagnóstico e tratamento da MAC por macrorregião do Brasil.

**Métodos:**

Estudo ecológico de dados disponíveis de 2000 a 2015. Informações sobre NV, óbitos e ecocardiógrafos foram obtidas do DATASUS, o IDH, do *Atlas de Desenvolvimento Humano no Brasil*, e as demais foram obtidas da demografia médica. Foram realizadas medidas de correlação entre as variáveis utilizando o índice de Kendall.

**Resultados:**

A taxa de MC foi 660,8/100.000 NV, das quais, 18.444 por MAC (taxa de diagnóstico 38,55/100.000 NV). As regiões Sul e Sudeste, com maiores valores de IDH e recursos, apresentaram as maiores taxas de diagnóstico de MAC (56,94/100.000 e 62,83/100.000 NV, respectivamente). As regiões Norte e Nordeste, com os menores valores de IDH e recursos, apresentaram as menores taxas de diagnóstico de MAC (9,77/100.000 e 13,43/100.000 NV, respectivamente). Essa taxa de diagnóstico foi 6,4 vezes maior no Sudeste do que no Norte, mas as taxas de mortalidade foram similares.

**Conclusão:**

Das MC, as MAC apresentaram a maior mortalidade nos menores de 1 ano no Brasil.

## Introdução

Define-se malformação congênita (MC) como a formação defeituosa de um órgão ou conjunto de órgãos que determina a presença de anormalidade morfológica ao nascimento.^1^ Em 2015, foram diagnosticados 2.368 portadores de MC por 100.000 nascidos vivos (NV) em todo o mundo, uma taxa de 7,6%, dos quais 10,6% morreram no primeiro ano de vida e 43% devido a malformações do aparelho circulatório (MAC).^2^

A apresentação clínica das MAC varia desde ausência de sintomas relacionados à malformação até cardiopatia sintomática e risco de morte. Cerca de 25% de todas as MAC são classificadas como críticas e requerem intervenção no primeiro ano de vida, pois, em geral, são incompatíveis com a vida e altamente dependentes de adequado suporte médico hospitalar.^3^ A maioria das mortes por MAC pode ser evitada com o diagnóstico precoce e o tratamento adequado; portanto, as MAC são consideradas causas de óbito evitáveis.^4^

Em 2015, no Brasil, diagnosticou-se MC ao nascimento em 7,4% dos NV e as distribuições percentuais dos tipos de MC e mortalidade foram similares àquelas relatadas no estudo *Global Burden of Disease*.^2^ O Brasil é dividido em cinco macrorregiões geopolíticas (Norte, Nordeste, Centro-Oeste, Sudeste e Sul) bastante desiguais em termos de distribuição de recursos para atenção e acesso à saúde. Como essas desigualdades afetam os recursos dedicados ao diagnóstico e ao tratamento das MAC, é necessário investigar se tais desigualdades têm impacto no risco de morte por MAC no primeiro ano de vida.

Com base nessas observações, este estudo teve por objetivo avaliar a associação de diagnóstico de MAC ao nascimento e morte por MAC no primeiro ano de vida com o índice de desenvolvimento humano (IDH) e a disponibilidade de recursos humanos tecnológicos para o diagnóstico e tratamento de MAC por macrorregião brasileira.

## Métodos

Trata-se de estudo ecológico das associações do diagnóstico de MAC ao nascimento e da mortalidade por MAC no primeiro ano de vida com as seguintes variáveis: (1) distribuição de pediatras; (2) distribuição de cirurgiões cardiovasculares; (3) disponibilidade de centros de cirurgia cardíaca pediátrica; (4) número de ecocardiógrafos; e (5) IDH das macrorregiões brasileiras de 2000 a 2015.

Este estudo foi aprovado pelo Comitê de Ética em Pesquisa do Hospital Universitário Clementino Fraga Filho (número 44662215.4.0000.5257).

As informações referentes aos NV, assim como a presença de malformação ao nascimento, foram obtidas no Sistema de Informações sobre Nascidos Vivos (SINASC).^5^ Esse sistema contém informação proveniente de todas as declarações de nascidos vivos registrados no Brasil, em cada unidade federativa, e acha-se disponível em bases anuais fornecidas pelo banco de dados do Sistema Único de Saúde (DATASUS) (http://tabnet.datasus.gov.br/cgi/deftohtm.exe?sinasc/cnv/nvuf.def). O período estudado foi de 2000 a 2015, pois foi a partir de 2000 que pelo menos 95% das informações se tornaram disponíveis em sua completude, especialmente nas regiões Norte e Nordeste.

As informações referentes aos óbitos foram obtidas no site do DATASUS (http://datasus.gov.br/informacoes-de-saude/servicos2/transferencia-de-arquivos). Tais informações compreendem todas as declarações de óbito registradas no Brasil entre 2000 e 2015, ano a ano, por local de residência e por unidade federativa. De cada banco de dados, foram selecionados apenas os óbitos até um ano de vida incompleto.^5^

Os diagnósticos das malformações identificadas ao nascimento e os óbitos cujas causas básicas estão contempladas no capítulo XVII da CID-10 foram divididos em "MAC" e "outras malformações congênitas (OutMC)".^6^ De acordo com a localização anatômica da MC, as MAC foram distribuídas nas seguintes categorias: câmaras e septos cardíacos (Q20-21); valvas (Q22-23); outras (Q24 sem 9) e não especificadas (Q24.9); e vasos (Q25-28). As OutMC foram classificadas nas seguintes categorias: sistema nervoso (Q00-07); olho, ouvido face e pescoço (Q10-18); aparelho respiratório (Q30-34); fenda labial e palatina (Q35-37); aparelho digestivo (Q38-45); sistema geniturinário (Q50-56 e Q60-64); sistema osteomuscular (Q65-79); tegumento (Q80-85); outras malformações congênitas (Q86-89); anomalias cromossômicas (Q90-99); sífilis congênita (A50, capítulo I); e linfangioma e hemangioma (D18, capítulo II).

As distribuições de pediatras e cirurgiões cardiovasculares no país foram obtidas do estudo sobre demografia médica no Brasil realizado com base nos censos médicos de 2011, 2013 e 2015.^7-9^ As variáveis usadas para avaliar a rede de diagnóstico e tratamento das MAC foram obtidas no site do DATASUS e vêm sendo disponibilizadas mensalmente desde agosto de 2005. Essas variáveis consistiram em número de centros classificados como provedores de cirurgia cardiovascular pediátrica e número de ecocardiógrafos em uso. As admissões devidas a MAC em cada macrorregião têm sido disponibilizadas desde janeiro de 2000.^10^

O IDH de cada unidade federativa do ano 2010 foi obtido no *Atlas de Desenvolvimento Humano no Brasil*.^11^ O IDH de cada unidade federativa foi ponderado pelo tamanho da população de cada unidade para compor o IDH de cada macrorregião. As informações referentes às populações foram obtidas no site do Instituto Brasileiro de Geografia e Estatística (https://www.ibge.gov.br/apps/populacao/projecao), que fornece projeções para os anos 2000 a 2060.

Este estudo foi realizado utilizando bancos de dados oficiais disponíveis no site do DATASUS, que fornece informação anonimizada, que impede a identificação dos indivíduos.

### Análise estatística

Para cada macrorregião brasileira, calculamos as seguintes taxas referentes a crianças com menos de 1 ano: taxas de diagnóstico de MAC e de OutMC por 100.000 NV; taxas de mortalidade; e mortalidade proporcional por MAC específicas e por doenças do aparelho circulatório (DAC). As estimativas foram calculadas para cada ano e para o período total do estudo.

Os dados das variáveis usadas para avaliar as unidades de saúde capacitadas para o diagnóstico e tratamento de MAC não estavam disponíveis para todo o período do estudo. Logo, calculou-se uma estimativa anual desses dados usando-se a média dos períodos disponíveis. Os indicadores correspondendo a cada uma das variáveis, com os números de NV usados como denominadores, foram calculados por macrorregião. Os coeficientes usados para pediatras, cirurgiões cardiovasculares, centros de cirurgia pediátrica cardiovascular e ecocardiógrafos foram calculados em relação a 1.000 NV e os coeficientes para hospitalização foram calculados em relação a 100.000 NV. Medidas de correlação foram realizadas usando-se o índice de Kendall, com significância de 95%.

Os dados obtidos no DATASUS foram analisados com Stata,^12^ versão 14 (StataCorp LP, College Station, TX, EUA), em tabelas exportadas para planilhas (Microsoft Excel, Microsoft, Seattle, WA, EUA).^13^

## Resultados

Entre 2000 e 2015 foram registrados 47.715.968 NV no Brasil. A presença de MC em algum órgão ou sistema foi detectada em 315.322 (0,66%) nascimentos, resultando em uma taxa de 660,8/100.000 NV. De todas as MC, 18.444 eram MAC, com taxa de diagnóstico de 38,55/100.000 NV.

**Figura 1:**
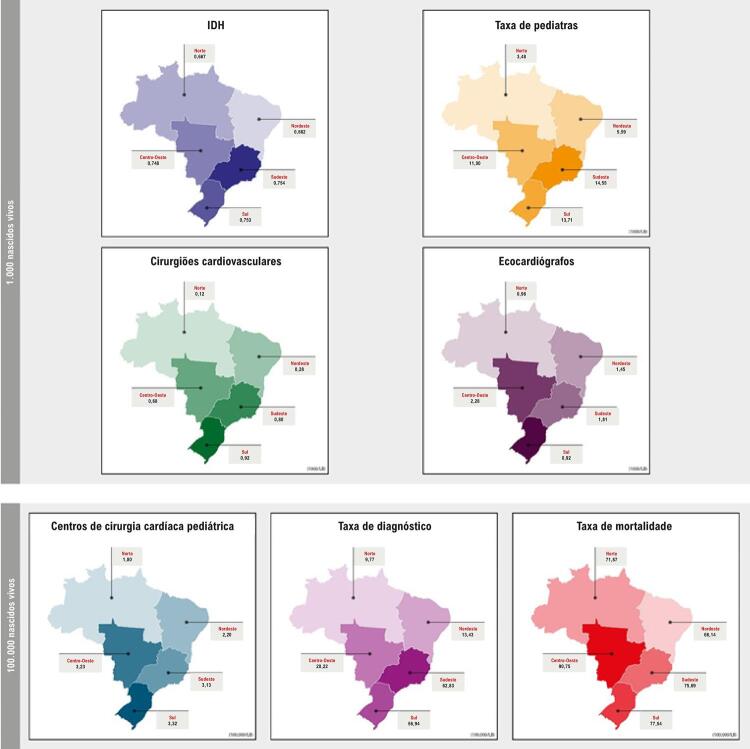
– Recursos tecnológicos e humanos para diagnóstico e tratamento de malformações do aparelho circulatório por macrorregião brasileira. IDH: índice de desenvolvimento humano.

**Figura 2:**
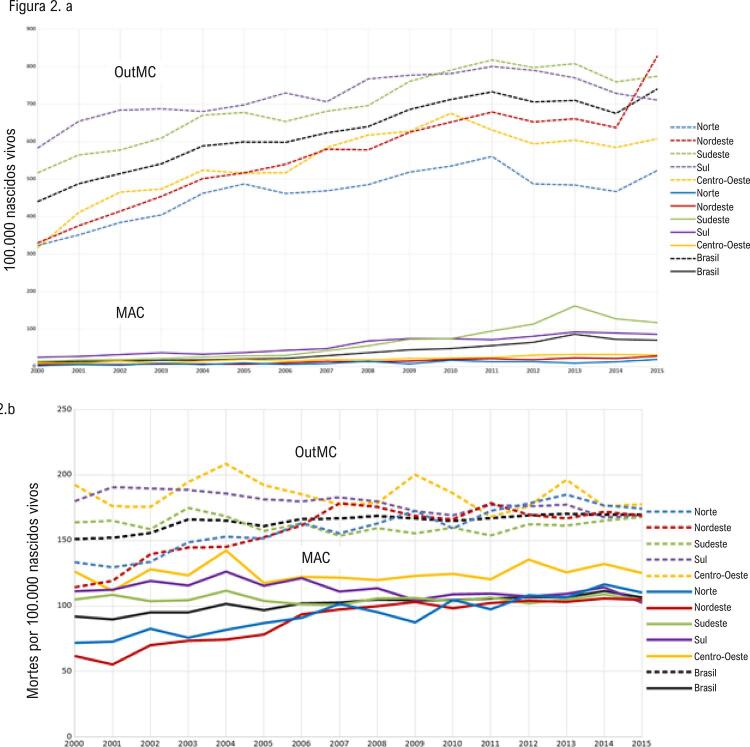
– A) Diagnóstico ao nascimento de malformações do aparelho circulatório (MAC) e outras malformações congênitas (OutMC) em nascidos vivos no Brasil. B) Mortalidade por malformações do aparelho circulatório e outras malformações em menores de 1 ano no Brasil.

A distribuição da população brasileira por macrorregião foi a seguinte: 14,4% habitantes no Sul; 42,2% no Sudeste; 8,3% no Norte; 27,9% no Nordeste; e 7,3% no Centro-Oeste.^5^ As mais altas taxas de diagnóstico de MC por 100.000 NV foram observadas nas regiões Sul (719,47) e Sudeste (694,96), enquanto as regiões Norte (464,02), Centro-Oeste (546,73) e Nordeste (559,29) apresentaram taxas mais baixas com algumas oscilações ao longo do tempo. A Figura 1 mostra que as MAC diagnosticadas ao nascimento foram mais frequentes na região Sudeste (62,83/100.000 NV) e menos frequentes na região Norte (9,77/100.000 NV). As diferenças regionais nas taxas de diagnóstico de MAC tornaram-se maiores nos últimos 5 anos, especialmente devido à elevação das taxas de MAC na região Sudeste, enquanto as taxas de diagnóstico de OutMC aumentaram até 2011, com diferenças significativas entre as regiões, tendo se estabilizado desde então, exceto na região Nordeste (Figura 2a). Todas as correlações entre as variáveis em estudo foram positivas, isto é, aumentos ou reduções foram concomitantes em cada par de variáveis (Tabela 1).

**Tabela 1:** – Correlações das taxas de diagnóstico e de mortalidade de malformações do aparelho circulatório com índice de desenvolvimento humano (IDH), taxas de pediatras, de cirurgiões cardiovasculares, de ecocardiógrafos e de centros de cirurgia cardiovascular pediátrica (índice tau de Kendall).

	IDH	Taxa de pediatras	Cirurgiões cardiovasculares	Ecocardiógrafos	Centro de cirurgia pediátrica	Taxa de diagnóstico	Taxa de mortalidade
IDH	1,00						
Taxa de pediatras	0,80	1,00					
Cirurgiões cardiovasculares	0,60	0,80	1,00				
Ecocardiógrafos	0,40	0,60	0,80	1,00			
Centro de cirurgia pediátrica	0,40	0,60	0,80	1,00	1,00		
Taxa de diagnóstico	0,80	1,00	0,80	0,60	0,60	1,00	
Taxa de mortalidade	0,40	0,20	0,40	0,60	0,60	0,20	1,00

De 2000 a 2015, houve 756.201 mortes de menores de 1 ano no Brasil. Dessas, 126.877 ocorreram por MC em um órgão ou sistema, sendo a taxa de mortalidade de 119,76/100.000 NV. As MAC corresponderam a 38,2% dos óbitos por MC, com taxa de mortalidade de 73,98/100.000 NV. A Figura 2b mostra que as diferenças nas taxas de mortalidade por MAC entre as regiões diminuíram no período do estudo, com exceção da região Centro-Oeste, que manteve a mortalidade mais elevada em todo o período. O mesmo ocorreu com a mortalidade por OutMC, para a qual a região Centro-Oeste manteve as mais elevadas taxas em todo o período.

**Figura 3:**
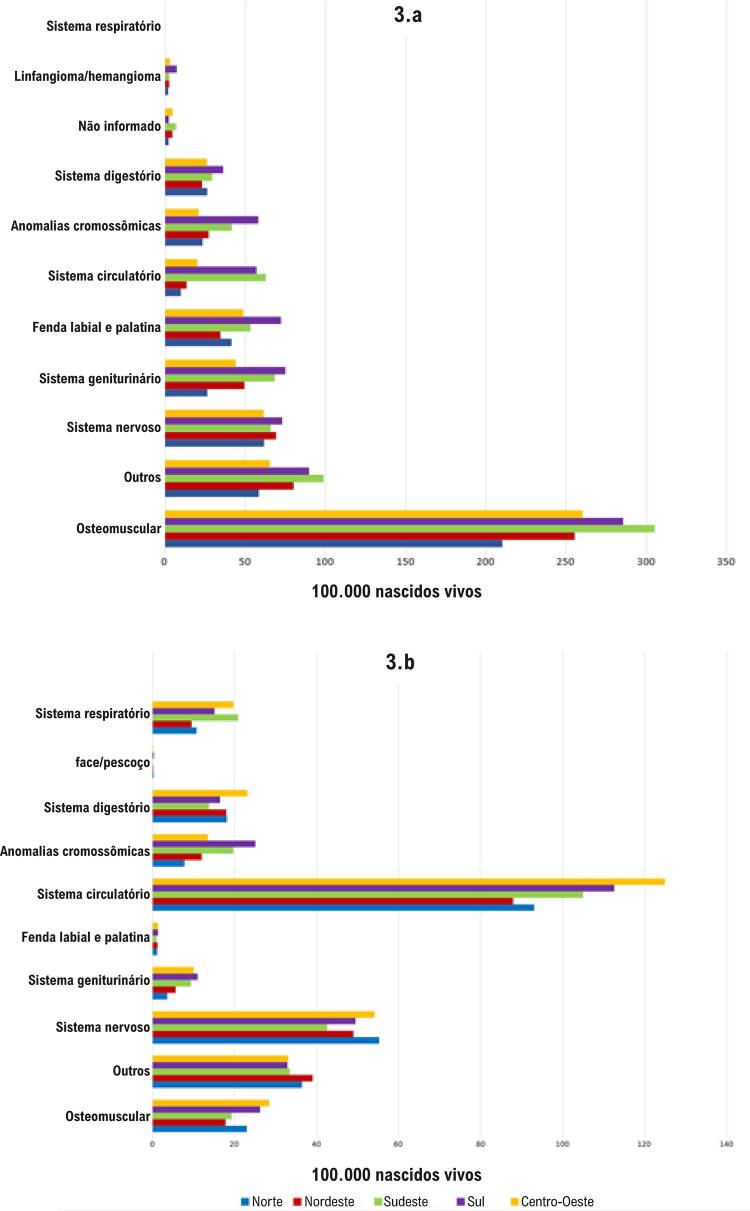
– A) Diagnóstico por grupos de tipos de malformações congênitas presentes ao nascimento entre 2000 e 2015, Brasil; B) Mortalidade infantil por grupos de tipos de malformações congênitas entre 2000 e 2015, Brasil.

Como ilustrado na Figura 3a, em todas as regiões, o tipo de MC por órgão ou conjunto de órgãos afetados mais frequentemente diagnosticado ao nascimento foi "malformação do sistema osteomuscular" (41,5%), seguida de "outras malformações congênitas" (10,1%). As MAC corresponderam a 5,9% de todos os diagnósticos de MC, sendo que 63,8% dos diagnósticos de MAC ocorreram na região Sudeste. Vale ressaltar que a região Sudeste apresentou 39,2% dos NV no Brasil no período estudado, sendo a distribuição de NV nas outras regiões a seguinte: 10,71% no Norte; 29,02% no Nordeste; 13,07% no Sul; e 7,86% no Centro-Oeste. A Figura 3b mostra que de 2000 a 2015, no Brasil, as MAC foram a principal causa de morte por MC (38,2%), enquanto as malformações do sistema osteomuscular corresponderam a 7,9% dessas mortes. A região Centro-Oeste apresentou a maior taxa de mortalidade atribuída a MAC (90,75/100.000 NV) no mesmo período.

A Tabela 2 mostra a mortalidade proporcional em menores de 1 ano atribuída a DAC e MAC, com subdivisão por causas específicas. Mais de 83% das mortes por causas do aparelho circulatório foram devidas a MAC em todas as regiões, com ênfase na região Sul, onde aquela porcentagem foi de 93%. Nesse conjunto de DAC e MAC, as MAC não especificadas corresponderam a metade das mortes dos menores de 1 ano no Brasil, das quais menos da metade (44%) ocorreu apenas na região Sudeste. A mortalidade proporcional por DAC foi 2,5 vezes maior na região Norte do que na região Sul. Cardiomiopatia representou 32% das mortes por DAC, emergindo como a principal causa de morte nesse subconjunto em todas as regiões.

**Tabela 2:** – Mortalidade proporcional por doenças do aparelho circulatório e malformações do aparelho circulatório em menores de 1 ano entre 2000 e 2015 subdividida por causa específica e distribuída por macrorregião brasileira

Causas de morte		Norte	Nordeste	Sudeste	Sul	Centro-oeste	Total
Cardiopatia pulmonar e doença da circulação pulmonar	Mortes MP (%)	84 1.51	189 1.31	396 1,76	62 0,83	76 1,49	807 1,46
Pericardite e endocardite	Mortes MP (%)	27 0,49	60 0,41	142 0,63	21 0,27	27 0,53	277 050
Miocardite	Mortes MP (%)	10 0,18	25 0,17	84 0,37	49 0,65	8 0,16	176 0,32
Cardiomiopatias	Mortes MP (%)	277 4,99	713 4,93	933 4,14	122 1,63	171 3,36	2216 4,02
Insuficiência cardíaca	Mortes MP (%)	163 2,94	347 2,40	276 1,22	73 0,97	56 1,10	915 1,66
Doenças cerebrovasculares e de outros vasos	Mortes MP (%)	121 2,18	249 1,72	459 2,03	66 0,88	83 1,63	978 1,77
Outras doenças do aparelho circulatório	Mortes MP (%)	240 4,32	541 3,74	648 2,87	97 1,29	120 2,36	1646 2,98
**Subtotal DAC**	**Mortes MP (%)**	**922 16,61**	**2124 14,68**	**2938 13,02**	**490 6,53**	**541 10,63**	**7015 12,71**
Câmaras e septos	Mortes MP (%)	670 12,07	1554 10,74	4260 18,88	1357 18,08	866 17,01	8707 15,78
Válvulas	Mortes MP (%)	172 3,10	428 2,96	1605 7,11	699 9,31	282 5,54	3186 5,77
Não especificada	Mortes MP (%)	2927 52,74	8283 57,25	9902 43,89	3834 51,08	2759 54,20	27705 50,21
Outras	Mortes MP (%)	531 9,27	1199 8,29	1600 7,09	384 5,12	254 4,99	3968 7,19
Vasos	Mortes MP (%)	328 5,91	81 6,09	2255 9,99	742 9,88	388 7,62	4594 8,33
**Subtotal MAC**	**Mortes MP (%)**	**4628 83,39**	**12345 85,32**	**19622 86,98**	**7016 93,47**	**4549 89,37**	**48160 87,29**
**Total DAC + MAC**	**Mortes MP (%)**	**5550 100,00**	**14469 100,00**	**22560 100,00**	**7506 100,00**	**5090 100,00**	**55175 100,00**

*MP: mortalidade proporcional; DAC: doenças do aparelho circulatório; MAC: malformações do aparelho circulatório; total DAC + MAC: total de mortes por distúrbios do aparelho circulatório*

Entre 2000 e 2015, a taxa de diagnóstico de MAC ao nascimento foi 6,4 vezes maior no Sudeste do que no Norte, mas as taxas de mortalidade foram similares nas duas macrorregiões. Comparado ao Sudeste, o Centro-Oeste teve quase a mesma quantidade de recursos (pediatras, cirurgiões cardiovasculares, centros com cirurgia pediátrica cardiovascular e número de ecocardiógrafos) mas realizou 3 vezes menos diagnósticos de MAC e apresentou taxa de mortalidade 1,2 vez maior (Figura 1).

## Discussão

Nas cinco macrorregiões brasileiras, entre 2000 e 2015, foram identificados três padrões de associação do diagnóstico de MAC ao nascimento e de morte por MAC com IDH e recursos tecnológicos e humanos dedicados ao diagnóstico e tratamento de MAC. As regiões Sul e Sudeste, com os maiores valores de IDH e recursos tecnológicos e humanos, apresentaram as maiores taxas de diagnóstico de MAC ao nascimento e de morte por MAC, esta última consequente à maior capacidade diagnóstica. As regiões Norte e Nordeste, com os mais baixos valores de IDH e recursos, apresentaram as mais baixas taxas de diagnóstico de MAC ao nascimento e morte por MAC no primeiro ano de vida. Entretanto, a região Centro-Oeste, com recursos similares aos das regiões Sul e Sudeste, apresentou baixa taxa de diagnóstico de MAC ao nascimento e melhora no diagnóstico de MAC por ocasião da morte.

Comparando as taxas de diagnóstico de MC e de MAC ao nascimento no Brasil com as taxas globais, a partir de dados do estudo *Global Burden of Disease*, no mesmo período e na mesma faixa etária do nosso estudo, observamos que nossas taxas de diagnóstico de MC e MAC foram 1,40 e 1,78 vez menor, respectivamente, do que as taxas globais.^2^ Mesmo na região Sul, que apresentou taxas de diagnóstico mais altas do que as demais regiões, as taxas de diagnóstico de MC e MAC foram 1,30 e 1,70 vez menor, respectivamente, em comparação às taxas globais. As taxas da região Norte, em comparação às de países da América do Norte, Europa Ocidental, Austrália e Japão, foram ainda mais baixas: 1,42 vez menor para MC e 1,83 vez menor para MAC.^2,14^

A diferença nas taxas de diagnóstico de MAC entre os países acha-se relacionada às variações em investimento em recursos de atenção à saúde. Países com maiores valores de produto interno bruto apresentam taxas de diagnóstico de MAC mais altas.^15,16^ Relações similares foram encontradas com o IDH e a taxa de diagnóstico de MAC entre as regiões estudadas e os países que possuíam IDH similar.^2^ Caneo et al.,^16^ compararam a necessidade de cirurgia cardíaca e o número dessas cirurgias realizadas no primeiro ano de vida em 2010 entre o estado de São Paulo e a Polônia, que tinham IDH e tamanho populacional similares na ocasião.^16^ Aqueles autores relataram que, em crianças menores de 1 ano na Polônia, 100% das cirurgias necessárias foram realizadas, enquanto, no estado de São Paulo, apenas 50%. Isso pode ser explicado pela disponibilidade de acesso a recursos de saúde, que ocorre de maneira heterogênea no Brasil devido a diferenças geográficas, socioculturais e econômicas.^16-18^

Entre 2000 e 2015, houve aumento nas taxas de diagnóstico de OutMC e MAC ao nascimento em todas as regiões brasileiras, tendo as diferenças percentuais permanecido constantes no período. Nas regiões Sul e Sudeste, a redução nas mortes foi acompanhada por uma melhora nas taxas de diagnóstico ao longo do tempo, enquanto nas regiões Norte e Nordeste, houve uma melhora na habilidade para diagnóstico no período próximo ao óbito, o que resultou em um aumento da taxa de mortalidade tanto por OutMC quanto por MAC. A região Sudeste, com as maiores taxas de diagnóstico de MAC ao nascimento, apresentou taxa de mortalidade por MAC similar àquela da região Norte; portanto, em comparação à região Norte, os recursos na região Sudeste conseguiram evitar mortes por MAC ao nascimento. Ao longo do período estudado, as diferenças entre as taxas de mortalidade por OutMC e por MAC nas regiões Norte e Nordeste diminuíram na comparação com as das demais regiões. Tal fato pode ser atribuído ao “Pacto pela Redução da Mortalidade Materna e Neonatal”,^19^ firmado em 2004 entre os três níveis federativos de atenção à saúde no Brasil. As estratégias de obtenção dessa meta foram traçadas para diminuir a mortalidade com mais ênfase nas regiões Norte e Nordeste, resultando em maior aporte de recursos e de pessoal para essas regiões. É provável que o aclive acentuado na curva de OutMC entre 2014 e 2015 na região Nordeste tenha sido causado pelo surto do Zika vírus, que contribuiu para malformações do sistema nervoso.^20^

As regiões Norte e Nordeste possuem maior percentual de diagnóstico de óbito por DAC no primeiro ano de vida, o que pode ser explicado pela confusão entre os diagnósticos de MAC e de DAC, provavelmente mais frequente nessas regiões, pois o diagnóstico diferencial é mais difícil entre essas causas quando os recursos diagnósticos são escassos ou tardios.^21^

Dentre as MC, as MAC apresentam maior mortalidade, pois necessitam de diagnóstico precoce para instituição do tratamento, visto que, das crianças que nascem com alguma cardiopatia congênita sem intervenção médica, 14% não sobrevivem ao primeiro mês de vida e 30% ao primeiro ano.^22^ Intervenção cirúrgica no primeiro ano de vida é necessária em 50% dos portadores de cardiopatia congênita, o que demanda complexidade de recursos técnico e humano, representando elevado custo de tratamento.^23-25^ No entanto, para o adequado suporte terapêutico, é necessário o correto diagnóstico da MAC e, no Brasil, mesmo nas regiões Sul e Sudeste, as MAC não especificadas são as mais frequentes, refletindo não só dificuldade no acesso como baixa qualidade de diagnóstico.

O número de ecocardiógrafos mostrou fraca relação com a taxa de diagnóstico de MAC por macrorregião. A região Norte apresentou a menor relação entre número de aparelhos disponíveis e taxas de diagnóstico; porém, a região Centro-Oeste, que possuía quantidade de aparelhos semelhante à da região Sul, realizou três vezes menos diagnósticos. O Sudeste, que possuía menor relação de ecocardiógrafos que o Centro-Oeste, realizou 3,1 vezes mais diagnósticos. Isso pode ser explicado pelo fato de que nem todos os aparelhos em uso no Brasil podem ser utilizados para a realização de exames neonatais e pediátricos, dependendo da disponibilidade de sondas adequadas à faixa pediátrica, do *software* específico, do treinamento do ecocardiografista em exames pediátricos, da presença do aparelho próximo aos locais de maior demanda e da acessibilidade dos pacientes.^26-28^

O número de cirurgiões cardiovasculares, de pediatras e de serviços de cirurgia cardíaca pediátrica segue a mesma relação que o número de ecocardiógrafos, demonstrando não ser apenas a quantidade de recursos técnicos e humanos por região que determina sua capacidade diagnóstica, mas de como e quando esses recursos são disponibilizados para a população. O diagnóstico precoce das MC, incluindo as MAC, deveria ocorrer no período pré-natal para possibilitar o encaminhamento dos pacientes para centros especializados de tratamento, mesmo antes do nascimento.^29,30^

Uma das limitações deste estudo, foi o uso de bancos de dados oficiais, cujas informações foram coletadas por instituições que assistem ao parto e ao óbito e que não seguem um protocolo único nem um planejamento de pesquisa. Outra limitação foi a análise de dados de fontes com diferentes disponibilidades temporais. Entretanto, esse problema não afetou nossos resultados, pois os dados apresentaram pouca oscilação no período estudado e as médias calculadas foram similares àquelas dos anos seguintes. Outra limitação foi a heterogeneidade da qualidade da informação sobre as causas de óbito e diagnóstico de MC nos certificados de óbito e de nascidos vivos, que requerem preenchimento adequado e adesão às regras da CID-10. No entanto, os certificados de óbito e de nascidos vivos são as únicas fontes abrangentes disponíveis de dados sobre óbito e nascimento no Brasil que permitem análises populacionais. Outra possível limitação foi o uso apenas do diagnóstico de MAC ao nascimento, uma vez que muitas MC só são diagnosticadas nos primeiros dias de vida; além disso, a ausência de informação sobre o momento em que o diagnóstico foi feito não permitiu que se conhecesse o tempo decorrido entre o diagnóstico e a morte por MAC.

## Conclusão

As diferenças regionais nas taxas de diagnóstico de MAC ao nascimento e de morte por MAC no Brasil acham-se associadas à desigualdade de disponibilidade de recursos tecnológicos e humanos para atenção à saúde. Quanto menor a disponibilidade desses recursos, menos se diagnostica ou maior é o atraso em se fazer o diagnóstico. A subestimação das taxas de morte por MAC nas regiões Norte e Nordeste deveu-se à concorrência com outras causas, como DAC, causas externas e mal definidas, assim como deficiências no diagnóstico. Diversas ações são necessárias para melhorar o diagnóstico e o tratamento das MAC, como medidas para ampliar os recursos de atenção à saúde, cujo acesso deve ser disponibilizado para toda a população.
