# Supplementary material for: IDH, Recursos Tecnológicos e Humanos para Diagnóstico e Tratamento das Malformações do Aparelho Circulatório no Brasil
Source: Arq Bras Cardiol. 2021 Jul 15;117(1):63–71. [Article in Portuguese] doi: 10.36660/abc.20200179 (PMC8294747; doi:10.36660/abc.20200179)
Supplement: Translation

# Translation: HDI, Technological and Human Resources in the Diagnosis and Treatment of Malformations of the Circulatory System in Brazil

**Authors:** Thais Rocha Salim, Thayanne Mendes Andrade, Carlos Henrique Klein, Gláucia Maria Moraes de Oliveira

**Affiliations:** 1 BrasilUniversidade Federal do Rio de Janeiro, Rio de Janeiro, RJ - Brasil; 2 BrasilUniversidade de Vassouras, Vassouras, RJ - Brasil; 3 BrasilEscola Nacional de Saúde Pública, Rio de Janeiro, RJ - Brasil

**Keywords:** Congenital Abnormalities, Cardiovascular Abnormalities, Heart Defects, Congenital/diagnosis, Infant Mortality, Epidemiology

## Abstract

**Background:**

In 2015, the number of infants with congenital malformations (CMs) per 100 000 live births (LBs) was 2368 (7.6%) worldwide, of whom 10.6% died in the first year of life, 43% due to malformations of the circulatory system (MCSs), a scenario similar to what occurs in Brazil.

**Objective:**

To assess, per Brazilian macroregion, whether diagnosis of MCS at birth and death due to MCS in the first year of life associate with human development index (HDI) and with technological and human resources.

**Methods:**

Ecological study including data available in 2000–2015. Data of LBs, deaths and availability of echocardiography devices were obtained from the DATASUS website. The HDI was obtained from the Atlas of Human Development in Brazil, while other variables were obtained from medical demographic data. Correlation measures between the variables were performed using the Kendall index.

**Results:**

The CM rate was 660.8/100 000 LBs, of which 18 444 were due to MCS (diagnosis rate, 38.55/100 000 LBs). Of all Brazilian macroregions, the Southern and Southeastern regions, with the highest HDI values and resources, had the highest MCS diagnosis rates (56.94/100 000 and 62.83/100 000 LBs, respectively). The Northern and Northeastern regions, with the lowest HDI values and resources, had the lowest MCS diagnosis rates (9.77/100 000 and 13.43/100 000 LBs, respectively). The MCS diagnosis rate was 6.4-fold higher in the Southeastern region as compared to the Northern region, but mortality rates were similar in both regions.

**Conclusion:**

Of the CMs, the MCS accounted for the highest number of deaths in children under the age of 1 year in Brazil.

## Introduction

Congenital malformation (CM) is defined as any defect in the formation of an organ or set of organs that determines the presence of a morphological abnormality at birth.^1^ In 2015, the number of infants with CMs per 100 000 live births (LBs) worldwide was 2368 (7.6%), of whom 10.6% died in the first year of life, 43% due to malformations of the circulatory system (MCSs).^2^

The clinical presentation of MCSs varies from absence of malformation-related symptoms to symptomatic cardiac disease and risk of death. Approximately 25% of all MCSs are deemed critical and require intervention in the first year of life because they are often incompatible with life and highly dependent on adequate medical and hospital support.^3^ Most deaths due to MCSs could be prevented with early diagnosis and appropriate treatment; therefore, MCSs are considered to be preventable causes of death.^4^

In 2015 in Brazil, 7.4% of the LBs were diagnosed with some CM at birth, and the percentage distributions of the types of CM and mortality were similar to those reported in the *Global Burden of Disease Study*.^2^ Geopolitically, Brazil is subdivided into five “macroregions” (North, Northeast, West-Central, Southeast, and South), highly unequal in terms of distribution of resources and access to health care. Since those inequalities affect resources dedicated to the diagnosis and treatment of MCSs, it is important to investigate whether those inequalities impact the risk of death from MCSs in the first year of life.

Based on the above observations, the aim of this study was to assess the association of the diagnosis of MCS at birth and death from MCS in the first year of life with the human development index (HDI) and the availability of technological and human resources dedicated to the diagnosis and treatment of MCSs per Brazilian macroregion.

## Methods

Ecological study of the associations of MCS diagnosis at birth and MCS mortality in children younger than 1 year of age with (1) distribution of pediatricians, (2) distribution of cardiovascular surgeons, (3) availability of centers providing pediatric cardiac surgery, (4) number of echocardiography devices, and (5) HDI of the Brazilian macroregions, from 2000 to 2015. The study was approved by the Research Ethics Committee of the Clementino Fraga Filho University-affiliated Hospital (number 44662215.4.0000.5257).

Data regarding LBs, as well as the presence of malformations at birth, were obtained from the Brazilian Information System on Live Births (in Portuguese, *Sistema de Informações sobre Nascidos Vivos* - SINASC,),^5^ which contains information of all live birth certificates registered in each Brazilian federative unit and is available on annual datasets from the Brazilian Unified Health System database (DATASUS) (http://tabnet.datasus.gov.br/cgi/deftohtm.exe?sinasc/cnv/nvuf.def). We chose to study the period between 2000 and 2015 because of the completeness of information available since 2000, especially in the Northern and Northeastern regions.

Death-related data were obtained from the DATASUS website (http://datasus.gov.br/informacoes-de-saude/servicos2/transferencia-de-arquivos). These sets of information comprise a combination of all death certificates registered in Brazil between 2000 and 2015, year by year, per place of residence, and per federative unit. From each database, we selected only deaths of children under the age of 1 year.^5^

The malformations identified at birth and the deaths whose underlying causes related to the ICD-10 chapter XVII were subdivided into “MCSs” and “other congenital malformations (OthCMs)”.^6^ According to the anatomic location of the malformation, MCSs were distributed into the categories “cardiac chambers and septa” (Q20-21), “valves” (Q22-23), “others” (Q24 except 9) and “unspecified” (Q24.9), and “vessels” (Q25-28). The OthCMs were classified as follows: “nervous system”(Q00-07); “eye, ear, face, and neck” (Q10-18); “respiratory system” (Q30-34); “cleft lip and palate” (Q35-37); “digestive system” (Q38-45); “genital organs and urinary system” (Q50-56 and Q60-64); “musculoskeletal system” (Q65-79); “integument” (Q80-85); “other congenital malformations” (Q86-89); “chromosomal abnormalities” (Q90-99); “congenital syphilis” (A50, chapter I); and “lymphangioma and hemangioma” (D18, chapter II).

The distributions of pediatricians and cardiovascular surgeons across the country were obtained from the study on medical demography based on the medical censuses of the years 2011, 2013, and 2015.^7-9^ The variables used to assess the healthcare for the diagnosis and treatment of MCS were obtained from the DATASUS website and have been made available monthly since August 2005. Those variables included the number of centers classified as providing pediatric cardiovascular surgery and the number of echocardiography devices in use. Admissions due to MCSs in each macroregion have been made available from January 2000 onwards.^10^

The HDI of each federative unit for the year 2010 was obtained from the *Atlas of Human Development in Brazil*.^11^ The HDI of each federative unit was weighted by each unit’s population size to yield the HDI of each macroregion. Information regarding the populations was obtained from the website of the Brazilian Institute of Geography and Statistics (https://www.ibge.gov.br/apps/populacao/projecao), which provides projections for the years 2000 to 2060.

This study was carried out using official databases, available on the DATASUS website, which provide anonymized information, thus not allowing the identification of the individuals.

### Statistical analysis

For each Brazilian macroregion, we calculated the following rates related to children under the age of 1 year: (A) diagnostic rates of MCSs and OthCMs per 100 000 LBs, (B) mortality rates, and (C) proportional mortality from specific MCSs and from circulatory system diseases (CSDs). The estimates were calculated for each year and for the entire study period.

Data of the variables used to assess the healthcare units providing the diagnosis and treatment of MCSs were not available for the entire study period. Therefore, we calculated an annual estimate of these data using the mean of the available periods. The indicators corresponding to each of these variables, with the numbers of LBs used as denominators, were calculated by macroregion. The coefficients used for pediatricians, cardiovascular surgeons, centers providing pediatric cardiovascular surgery, and echocardiographic devices were calculated in relation to 1000 LBs and the coefficients for hospitalizations were calculated in relation to 100 000 LBs. Correlation measures were performed using the Kendall index, with a 95% significance index.

The data obtained from the DATASUS were analyzed with Stata,^12^ version 14 (StataCorp LP, College Station, TX, USA), in tables exported to spreadsheets (Microsoft Excel, Microsoft, Seattle, WA, USA).^13^

## Results

A total of 47 715 968 LBs were registered in Brazil between 2000 and 2015. The presence of CM in any organ or system was detected in 315 322 (0.66%) births, resulting in a rate of 660.8/100 000 LBs. Of all CMs, 18 444 were MCSs, with a rate of diagnosis of 38.55/100 000 LBs.

The distribution of the Brazilian population by macroregion is as follows: 14.4% inhabitants in the Southern region, 42.2% in the Southeastern, 8.3% in the Northern, 27.9% in the Northeastern, and 7.3% in the West-Central region.^5^ The highest rates of diagnosis of CMs per 100 000 LBs occurred in the Southern (719.47) and Southeastern (694.96) regions, while the Northern (464.02), West-Central (546.73) and Northeastern regions (559.29) showed lower rates with some oscillations over time. As shown in Figure 1, MCSs diagnosed at birth were more frequent in the Southeastern region (62.83/100 000 LBs) and less frequent in the Northern region (9.77/100 000 LBs). Regional differences in the rates of diagnosis of MCSs have become wider in the last 5 years, especially due to higher rates of MCSs in the Southeastern region, while diagnostic rates of OthMCs increased until 2011, with reasonable differences between regions, and stabilized from then on, except for the Northeastern region (Figure 2a). All correlations between the variables studied were positive, that is, increases or decreases were concomitant in each pair of variables (Table 1).

**Figure 1:** – Technological and human resources for the diagnosis and treatment of malformations of the circulatory system by Brazilian macroregion. HDI: human development index; LB: live births

**Figures 2:** – A) Diagnosis at birth of malformations of the circulatory system (MCS) and other congenital malformations (OthCM) in live births in Brazil. B) Mortality due to malformations of the circulatory system and other congenital malformations in children younger than 1 year, Brazil.

**Table 1:** – Correlations of diagnostic and mortality rates of malformations of the circulatory system with human development index (HDI) and rates of pediatricians, of cardiovascular surgeons, of echocardiographic devices and of pediatric cardiac surgery centers (Kendall index)

	HDI-M	Rate of pediaticians	Cardiovascular surgeons	Echocardiography devices	Pediatric cardiac surgery centers	diagnostic rate	Mortality rate
HDI-M	1.00						
Rate of pediatricians	0.80	1.00					
Cardiovasuclar surgeons	0.60	0.80	1.00				
Echocardiography devices	0.40	0.60	0.80	1.00			
Pediatric cardiac surgery centers	0.40	0.60	0.80	1.00	1.00		
Diagnostic rate	0.80	1.00	0.80	0.60	0.60	1.00	
Mortality rate	0.40	0.20	0.40	0.60	0.60	0.20	1.00

In the period from 2000 to 2015, there were 756 201 deaths in children under the age of 1 year in Brazil. Of these, 126 877 occurred due to CM in any organ or system, with a mortality rate of 119.76/100 000 LBs. The MCSs corresponded to 38.2% of the deaths from CMs, with a mortality rate of 73.98/100 000 LBs. Figure 2b shows that the differences in the mortality rates from MCSs between the regions decreased over the study period, except for the West-Central region, where those rates remained the highest throughout the period. The same occurred with mortality due to OthCMs, in which the West-Central region remained with the highest rates throughout the period.

As shown in Figure 3a, the type of CM per organ or set of organs affected most frequently diagnosed at birth in all regions was “malformation of the musculoskeletal system” (41.5%) followed by “other congenital malformations” (10.1%). The MCSs corresponded to 5.9% of all CM diagnoses, and 63.8% of the MCS diagnoses occurred in the Southeastern region. It is worth noting that 39.2% of the LBs in the country in the study period occurred in the Southeastern region, while in other regions the distribution of LBs was as follows: 10.71%, in the North; 29.02%, Northeast; 13.07%, South; and 7.86% West-Central. Figure 3b shows that from 2000 to 2015, in Brazil, MCSs were the most frequent cause of death from CM (38.2%), while malformations of the musculoskeletal system corresponded to 7.9% of those deaths. The West-Central region had the highest mortality rate attributed to MCS (90.75/100 000 LBs) in the same period.

**Figure 3:** – A) Diagnosis by groups of types of congenital malformations present at birth between 2000 and 2015, Brazil; B) Childhood mortality by groups of types of congenital malformations between 2000 and 2015, Brazil.

Table 2 shows the proportional mortality in children under the age of 1 year attributed to CSD and MCS, with subdivision for specific causes. More than 83% of the deaths attributed to circulatory system causes were due to MCS in all regions, with emphasis on the Southern region, where that rate was 93%. In this set of CSD and MCS, unspecified MCSs accounted for half of the deaths of children under 1 year of age in Brazil, of which less than half (44%) occurred in the Southeast alone. The Northern region showed a proportional mortality due to CSD 2.5 times higher than that of the Southern region. Cardiomyopathy corresponded to 32% of the deaths from CSD and emerged as the main cause of death in this subset in all regions.

**Table 2:** – Proportional mortality from circulatory system diseases and malformations of the circulatory system in children younger than 1 year between 2000 and 2015 subdivided by specific cause and distributed by Brazilian macroregions

Cause of death		North	Northeast	Southeast	South	West-Central	Total
Pulmonary heart disease and disease of the pulmonary ciculation	Deaths PM (%)	84 1.51	189 1.31	396 1,76	62 0,83	76 1,49	807 1,46
Periocarditis and endocarditis	Deaths PM (%)	27 0,49	60 0,41	142 0,63	21 0,27	27 0,53	277 050
myocarditis	Deaths PM (%)	10 0,18	25 0,17	84 0,37	49 0,65	8 0,16	176 0,32
Cardiomyopathies	Deaths PM (%)	277 4,99	713 4,93	933 4,14	122 1,63	171 3,36	2216 4,02
Heart failure	Deaths PM (%)	163 2,94	347 2,40	276 1,22	73 0,97	56 1,10	915 1,66
Cerebrovascular diseases and diseases of other vessels	Deaths PM (%)	121 2,18	249 1,72	459 2,03	66 0,88	83 1,63	978 1,77
Outras doenças do aparelho circulatório	Deaths PM (%)	240 4,32	541 3,74	648 2,87	97 1,29	120 2,36	1646 2,98
**Subtotal DAC**	**Deaths PM (%)**	**922 16,61**	**2124 14,68**	**2938 13,02**	**490 6,53**	**541 10,63**	**7015 12,71**
Chambers and septa	Deaths PM (%)	670 12,07	1554 10,74	4260 18,88	1357 18,08	866 17,01	8707 15,78
Valves	Deaths PM (%)	172 3,10	428 2,96	1605 7,11	699 9,31	282 5,54	3186 5,77
Unspecified	Deaths PM (%)	2927 52,74	8283 57,25	9902 43,89	3834 51,08	2759 54,20	27705 50,21
Others	Deaths PM (%)	531 9,27	1199 8,29	1600 7,09	384 5,12	254 4,99	3968 7,19
Vessels	Deaths PM (%)	328 5,91	81 6,09	2255 9,99	742 9,88	388 7,62	4594 8,33
**Subtotal MCS**	**Deaths PM (%)**	**4628 83,39**	**12345 85,32**	**19622 86,98**	**7016 93,47**	**4549 89,37**	**48160 87,29**
**Total CSD + MCS**	**Mortes MP (%)**	**5550 100,00**	**14469 100,00**	**22560 100,00**	**7506 100,00**	**5090 100,00**	**55175 100,00**

*PM: proportional mortality; CSD: circulatory system disease; MCS: malformations of the circulatory system; total CSD + MCS: total deaths due to circulatory system disorders.*

Between 2000 and 2015, the rate of diagnosis of MCS at birth was 6.4 times higher in the Southeast than in the North, but the mortality rates were similar in both macroregions. Compared with the Southeast, the West-Central had almost the same amount of resources (pediatricians, cardiovascular surgeons, centers providing pediatric cardiovascular surgery, and number of echocardiography devices) but made 3 times less MCS diagnoses and had a 1.2-fold higher mortality rate (Figure 1).

## Discussion

Across the five Brazilian macroregions, between 2000 and 2015, we observed three patterns of association of the diagnosis of MCS at birth and death from MCS with HDI and technological and human resources dedicated to MCS diagnosis and treatment. The Southern and Southeastern regions, with the highest values of HDI and technological and human resources, showed the highest rates of diagnosis of MCSs at birth and of death attributed to MCSs, the latter consequent to better diagnostic ability. The Northern and Northeastern regions, with the lowest values of HDI and resources, had the lowest diagnostic rates of MCS at birth and death in the first year of life. On the other hand, the West-Central region, which had resources similar to those in the Southern and Southeastern regions, had a low rate of MCS diagnosis at birth and an improvement in the diagnosis of MCS by the time of death.

Comparing the diagnostic rates of CMs and MCSs at birth in Brazil with the global rates according to data provided by the *Global Burden of Disease Study* in the same period and age range of our study, we found the diagnostic rates of CM and MCS to be 1.40 and 1.78 times lower, respectively, than the global rates.^2^ Even in the Southern region, which performed better than the other Brazilian regions, the diagnostic rates for CMs and MCS were 1.30 times and 1.70 times lower, respectively, compared with the global rates. When comparing the rates of the Northern region with those of countries in North America, Western Europe, Australia, and Japan, ours were even lower: 1.42 times for CMs and 1.83 times for MCSs.^2,14^

The difference in the diagnostic rates of MCSs among countries is related to variations in investment in health care resources. Countries with higher gross domestic product and HDI have higher rates of MCS diagnosis.^15,16^ Similar relationships have been found with the HDI and the diagnosis rate of MCS among regions and countries with similar HDI.^2^ Caneo et al.^16^ have compared the need for cardiac surgery and the number of such surgeries performed in the first year of life in 2010 between the state of São Paulo and Poland, which had similar HDI and population size at that time.^16^ The authors have observed that, in children under the age of 1 year in Poland, 100% of the required surgeries were performed, whereas in the state of São Paulo, this rate was only 50%. This can be explained by the availability of access to health care resources, which occurs heterogeneously in Brazil due to geographical, sociocultural, and economic differences.^16-18^

Between 2000 and 2015, there was an increase in the diagnostic rates of OthCMs and MCS at birth in all Brazilian regions, and the percent differences between them remained constant in the period. In the Southern and Southeastern regions, the decrease in deaths was accompanied by an improvement in diagnostic rates over time, whereas in the Northern and Northeastern regions, there was an improvement in the diagnostic ability around the time of death, which resulted in an increased mortality rate for both OthCM and MCS. The Southeastern region, with the highest rates of MCS diagnosis at birth, showed death rates due to MCS similar to those of the Northern region; thus, as compared to the Northern region, the resources in the Southeastern region could more successfully prevent deaths from MCS at the time of birth. Over the study period, the differences in the mortality rates from OthCM and MCS in the Northern and Northeastern regions were reduced as compared to the other regions. This fact can be attributed to the results of the “Pact for the Reduction of Maternal and Neonatal Mortality”,^19^ signed in 2004 among the three Brazilian federative health care levels to reduce neonatal mortality. The strategies to achieve that goal were designed to reduce mortality with more emphasis on the Northern and Northeastern regions, resulting in a greater contribution of resources and personnel to those regions. In addition, the accentuated slope in the OthCM curve between 2014 and 2015 in the Northeastern region might have been caused by the outbreak of the Zika virus that contributed to malformations of the nervous system.^20^

The Northern and Northeastern regions had a higher percentage of diagnoses of deaths due to CSD in the first year of life, which can be explained by misdiagnoses of MCS and CSD, probably more frequent in those regions, because the differential diagnosis between those causes is more difficult when diagnostic resources are scarce or delayed.^21^

Of the CMs, MCSs had the highest mortality because they require an early diagnosis for therapy initiation, considering that among children born with some type of congenital heart disease, survival is below 14% during the first month of life and 30% during the first year in the absence of medical intervention.^22^ Surgical interventions in the first year of life are necessary for 50% of the patients with congenital heart disease, which results in high expenditures for treatment and complex technical and human resources.^23-25^ However, for adequate therapeutic support, a correct diagnosis of MCS is necessary, and, in Brazil, even in the Southern and Southeastern regions, unspecified MCSs are the most frequent CMs, which reflects not only difficulties in access to care but also poor quality of diagnosis.

The number of echocardiography devices showed a weak relation with the diagnosis rate of MCSs by macroregion. The Northern region had the lowest ratio between the number of available devices and diagnostic rates, but the West-Central region, which had a number of devices close to that of the Southern region, had a diagnosis rate 3 times lower. The Southeast, with a lower ratio of echocardiography devices than the West-Central, had a diagnosis rate 3.1 times higher. This can be explained by the fact that not all devices used in Brazil are able to perform neonatal and pediatric examinations, in addition to depending on the availability of probes appropriate for children, specific software, echocardiographers trained in pediatrics, devices located near sites with greater patient demand, and patient accessibility.^26-28^

The number of cardiac surgeons, pediatricians, and centers performing pediatric cardiac surgery followed the same relationship as the number of echocardiography devices, which demonstrates that it is not only the amount of technological and human resources per region that determines the diagnostic capacity, but also how and when these resources are made available to the population. Early diagnosis of CMs, including MCSs, should be established before birth to enable the patients to be referred to specialized treatment centers even before their birth.^29,30^

This study has limitations, such as the use of official databases, whose information had been collected by the institutions that provide assistance to childbirth and death and that do not follow a common protocol or research planning. Another limitation was the analysis of data from sources with temporal differences. However, this problem did not affect our results because the data underwent little variation over the study period and the calculated means were similar to those of the following years. Another limitation was the heterogeneous quality of the information about causes of death and diagnosis of CM in the death and live birth certificates, which depends on the adherence to the ICD-10 rules and the proper completion of death and live birth certificates. However, death and live birth certificates are the only comprehensive sources on Brazilian death and birth data available that allow population analysis. Other possible limitation was the use of the diagnosis of MCS only at birth, considering that many CMs are diagnosed over the first days of life. In addition, the time elapsed from diagnosis to death from MCS could not be known because information on the time of diagnosis was not available.

## Conclusion

Regional differences in the rates of diagnosis of MCS at birth and of death due to MCS in the first year of life in Brazil are associated with unequal availability of technological and human resources for health care. The lower that availability, the more delayed the diagnoses or the lower their number. The underestimation of death rates from MCS in the Northern and Northeastern regions was due to competition with other causes, such as CSD, external and ill-defined causes, as well as deficiencies in diagnosis. Several actions are necessary to improve the diagnosis and therapy of MCS, such as measures to increase the health care resources, whose access should be made available to the entire population.
